# Inhibition of hyperactivity of the dorsal raphe 5‐HTergic neurons ameliorates hippocampal seizure

**DOI:** 10.1111/cns.13648

**Published:** 2021-05-06

**Authors:** Heming Cheng, Yingbei Qi, Nanxi Lai, Lin Yang, Cenglin Xu, Shuang Wang, Yi Guo, Zhong Chen, Yi Wang

**Affiliations:** ^1^ Institute of Pharmacology & Toxicology College of Pharmaceutical Sciences Zhejiang University Hangzhou China; ^2^ Key Laboratory of Neuropharmacology and Translational Medicine of Zhejiang Province College of Pharmaceutical Science Zhejiang Chinese Medical University Hangzhou China; ^3^ Epilepsy Center Department of Neurology Second Affiliated Hospital School of Medicine Zhejiang University Hangzhou China

**Keywords:** deep brain stimulation, depression, dorsal raphe, epilepsy, serotonin

## Abstract

**Aims:**

Epilepsy, frequently comorbid with depression, easily develops drug resistance. Here, we investigated how dorsal raphe (DR) and its 5‐HTergic neurons are implicated in epilepsy.

**Methods:**

In mouse hippocampal kindling model, using immunochemistry, calcium fiber photometry, and optogenetics, we investigated the causal role of DR 5‐HTergic neurons in seizure of temporal lobe epilepsy (TLE). Further, deep brain stimulation (DBS) of the DR with different frequencies was applied to test its effect on hippocampal seizure and depressive‐like behavior.

**Results:**

Number of c‐fos^+^ neurons in the DR and calcium activities of DR 5‐HTergic neurons were both increased during kindling‐induced hippocampal seizures. Optogenetic inhibition, but not activation, of DR 5‐HTergic neurons conspicuously retarded seizure acquisition specially during the late period. For clinical translation, 1‐Hz‐specific, but not 20‐Hz or 100‐Hz, DBS of the DR retarded the acquisition of hippocampal seizure. This therapeutic effect may be mediated by the inhibition of DR 5‐HTergic neurons, as optogenetic activation of DR 5‐HTergic neurons reversed the anti‐seizure effects of 1‐Hz DR DBS. However, DBS treatment had no effect on depressive‐like behavior.

**Conclusion:**

Inhibition of hyperactivity of DR 5‐HTergic neuron may present promising anti‐seizure effect and the DR may be a potential DBS target for the therapy of TLE.

## Graphical abstract

Activity of the DR and its 5‐HTergic neurons remarkably increase during hippocampal kindling seizure. Either optogenetic inhibition of DR 5‐HTergic neurons or 1‐Hz DR DBS significantly retards seizure acquisition. The hyperactivity of the DR may serve as an underlying mechanism for propagation of seizure and the DR may be a potential DBS target for the treatment of epilepsy.

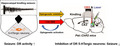

## 1 INTRODUCTION

Epilepsy is a serious brain disorder, afflicting nearly 1% of people worldwide. It is considered to be circuit‐level syndromes characterized by excessive hypersynchronous discharges due to the imbalance of excitatory‐inhibitory system in the brain.^1^ Temporal lobe epilepsy (TLE) has a relative higher drug‐resistant rate, of which the epileptogenic focuses are often located in the limbic systems, particularly the hippocampus.^2^ Patients with TLE not only suffer from recurrent epileptic seizures, but also the psychological, social, and cognitive consequences.^3^ Notably, depression is frequently comorbid in patients with TLE.^4^

Increasing number of evidence shows that the 5‐HTergic system plays a critical role not only in psychiatric function such as depression and anxiety, but also in epileptic seizures.^5^ Generally, depletion of brain 5‐HT or knock out 5‐HT_1a_ or 5‐HT_2c_ receptors increases seizure susceptibility.^6^, ^7^, ^8^, ^9^, ^10^ Conversely, increase of extracellular 5‐HT levels with fenfluramine or selective 5‐HT reuptake inhibitors (SSRIs) alleviates seizures,^11^, ^12^, ^13^ although exceptions have been described, too.^14^, ^15^ Even though most of antidepressants were considered to be safe for patients with epilepsy, several SSRIs were also reported with proconvulsant properties.^16^ Indeed, the 5‐HTergic neuronal system is divergent in anatomy, morphology, hodology, electrophysiology, and gene expression, which suggests functionally heterogeneous subtypes of 5‐HTergic neurons.^17^ Therefore, precise investigation of the function of 5‐HT subsystems in epilepsy is necessary.

The 5‐HTergic neurons are mainly located in various subregions of the raphe nuclei that distributed near the midline of the brainstem. The dorsal raphe nucleus (DR) is the predominant source of 5‐HTergic innervation of the forebrain,^18^ implicating in diverse functions including anxiety, depression, and sleep‐wake cycles.^19^, ^20^, ^21^ But functions of the DR in epilepsy are limited investigated. It is reported that neuronal activities of different raphe subregions in different epilepsy models are heterogeneous.^22^, ^23^ Even in the DR, population firing and identified 5‐HTergic neurons firing during seizure are diverse from each other.^24^, ^25^ What on earth the causal role of the DR and its 5‐HTergic system in TLE is still unknown. Early nonspecific interventions studies found that electrolytic lesion of the midbrain raphe enhanced the epileptiform activity in hippocampus kindling model and DBS of the median raphe (MR) significantly inhibited convulsion in different types of seizure models.^8^, ^26^, ^27^ However, a recent research indicated that activation of DR 5‐HTergic neurons in *DBA/1* mice reduced the respiratory arrest, but had limited effect on seizure itself.^28^ These results indicate that the DR may have an important but heterogeneous role in TLE and more direct evidence of the function of DR 5‐HTergic neurons in seizure should be provided. Here, by using calcium fiber photometry, optogenetics, and DBS, we investigate the role of DR and its 5‐HTergic neurons in epilepsy.

## 2 MATERIALS AND METHODS

### 2.1 Animals

Pet‐1 ETS gene is required for the differentiation of 5‐HTergic neurons.^29^ The *Pet‐cre* mice (stock No. 012712) were genotyped according to the protocols provided by Jackson Laboratory and were bred to C57BL/6J mice. Male *Pet‐cre* mice and C57BL/6J mice (at least 2 months old) were used. They were group housed with ad libitum access to standard pellet food and water in a 12/12 h light/dark cycle (lights on 8 a.m.). Mice were singly housed after surgeries to facilitate recovery. All behavior experiments were performed between 9:00 a.m. and 7:00 p.m., which were approved by the Zhejiang University Animal Experimentation Committee and were in complete compliance with the National Institutes of Health Guide for the Care and Use of Laboratory Animal and followed the ARRIVE guidelines.^30^

### 2.2 Viruses

For *in vivo* fiber photometry, 400 nl of AAV2/9‐Ef1α‐DIO‐GCaMP6m was injected into the DR of *Pet‐cre* mice. For optogenetic manipulations, 400 nl of AAV2/9‐Ef1α‐DIO‐eYFP or AAV2/9‐Ef1α‐DIO‐hChR2(H134R)‐eYFP or AAV2/9‐CAG‐FLEX‐ArchT‐GFP was injected into the DR of *Pet‐cre* mice. All viruses (viral titers >1.0 × 10^12^ particles/ml) were purchased from OBiO Technology Co., Ltd.

### 2.3 Stereotaxic surgery

Stereotaxic surgeries were performed as our previous studies.^31^, ^32^ Briefly, under sodium pentobarbital anesthesia (50 mg/kg, i.p.), mice were head‐fixed in a stereotaxic frame (512600, Stoelting) for injections and implantations with a heating pad. Virus (400 nl) was injected into the DR (AP: −4.4 mm; ML: ±0.0 mm; DV: −3.4 mm) at the rate of 100 nl/min with a 1‐μl microliter syringes controlled by pump (Micro4, World Precision Instruments). The virus was allowed to express for at least 3 weeks.

For *in vivo* fiber photometry and optogenetic manipulation in the hippocampal kindling epilepsy model, kindling electrodes made of twisted PFA‐coated stainless‐steel wires (791500; diameter, 0.127 mm; A‐M Systems) were implanted in right ventral hippocampus (CA3: AP: −2.8 mm; ML: −3.2 mm; DV: −3.2 mm) and affixed with dental cement for electrical stimulation and EEG recording. Then, an optical fiber attached to ceramic ferrule (200 μm, 0.37 NA, Inper Co. LTD) was implanted in the DR and affixed with dental cement to deliver light. Two screws were placed over the cerebellum to serve as the reference and ground electrodes.

For DBS of the DR in the kindling model, kindling electrodes were implanted in the right ventral hippocampus for electrical stimulation and EEG recording, and the DR for the delivery of DBS. After behavioral tests, the place of the electrode implantation and viral expression in all mice were verified and only the mice with correct place were taken into data analysis. These criteria were pre‐established.

### 2.4 Hippocampal kindling model

After one‐week surgery recovery, the CA3 of mice were stimulated with a constant‐current stimulator (SEN‐7203, SS‐202J; Nihon Kohden) and the EEGs were recorded with a Neuroscan system (NuAmps, Neuroscan System). The stimulation intensity was started at 40 μA and was then increased in 20‐μA steps every 1 min, until produced at least a 5‐s after‐discharge. This stimulation intensity was defined as after‐discharge threshold (ADT) of each mouse, which indicates epileptogenic sensibility for that mouse and was used for grouping thereafter. Subsequently, all mice received 10 kindling stimulations daily (400 μA, 20 Hz, 2‐s trains, 1‐ms monophasic square‐wave pulses). Seizure severity was classified into seizure stages 1–5 according to the Racine scale, among which stages 1–3 were considered as focal seizures (FS) and stages 4–5 as generalized seizures (GS).^33^ The severity of seizure was scored by a trained observer who was unaware of the experimental groupings.

### 2.5 In vivo fiber photometry

One week after surgery, fiber photometry was conducted during hippocampal kindling with the fiber photometry system (Nanjing Thinkertech) strictly according to our previous studies.^34^, ^35^ The GCaMP fluorescence was bandpass filtered and collected by a photomultiplier tube using a 488‐nm diode laser that coupled into a 200‐μm optical fiber. An amplifier was used to convert the photomultiplier tube current output to voltage signals, which was further filtered through a low‐pass filter (40 Hz cut‐off). We analyzed data based on events of individual trials of kindling stimulations and derived the values of fluorescence change (ΔF/F) by calculating (F−F0)/F0.

### 2.6 Light stimulation and DBS

473‐nm (20 Hz, 10 ms/pulse, 5 mW) or 589‐nm laser light (continuous, 5 mW) was delivered by the laser (IKECOOL Laser) through a 200‐μm optic fiber. The light stimulation was delivered immediately after kindling stimulation and stopped at the end of ADD manually.

To investigate the effect of DBS in the DR on the kindling model, mice were divided into four groups (sham, 1, 20, and 100 Hz). Mice in sham group were connected to the apparatus but no DBS was delivered while the other groups received DBS (monophasic square‐wave pulse, 0.1 ms per pulse) immediately after kindling through a constant‐current stimulator. The DBS threshold was 1/5 of the current intensity that induced abnormal behavior (300 μA for 1 Hz, 100 μA for 20 Hz, and 30 μA for 100 Hz).

### 2.7 Assessment of depressive‐like behavior

For all depressive‐like behavioral tests, mice were transported in a holding cabinet to the testing room with dim light (~20 lux) where they were habituated at least 1 day before testing.^36^ During the testing session, the behavior of each mouse was recorded by a video tracking system. Experiment and quantitative analyses were conducted by a trained experimenter who was unaware of the experimental groupings.

Sucrose preference test. Mice were housed individually and habituated with two identical bottles with 1% sucrose for 2 days, followed with 2‐day identical bottles with water. Then, the experimental mice were presented with two bottles (one containing water and the other containing 1% sucrose) for 2 days, and the bottles’ positions were switched after 1 day. The consumption of each fluid was measured, and sucrose preference was expressed as the percentage of (sucrose consumption)/(water consumption + sucrose consumption).

Tail suspension test. Each mouse was individually suspended 50 cm above the surface of a table using adhesive tape that was placed roughly 1 cm from the tip of the tail, which was videotaped from the side. Each mouse was tested only once for 6 min and the immobility time in the last 5 min was measured. Mice were considered immobile without initiated movements, including passive swaying.

Splash test. Each mouse was placed individually in their home cage and were sprayed with 10% sucrose solution on the back. The latency of grooming was recorded with a maximum of 5 min.

### 2.8 Immunohistochemistry

Mice were sacrificed and perfused with PBS and 4% PFA. After cryoprotected with 30% (w/v) sucrose, brains were cut with a sliding freezing microtome (NX70, Thermo Fisher Scientific) into 40‐μm coronal sections.

For c‐fos or TPH2 staining, sections were incubated at 4℃ overnight in mouse anti‐c‐fos (1:500, ab208942, Abcam) primary antibody or rabbit anti‐TPH2 (1:1000 dilution; NB100‐74555, Novus biologicals) primary antibody and donkey anti‐mouse Alexa 647 secondary antibody (1:1000; ab150107, Abcam) or donkey anti‐rabbit Alexa 647 (1:1000; ab150075, Abcam) secondary antibody for 2 h at room temperature.^37^ Slides were mounted with Dapi fluoromount media (Yeasen Biotech Co., Ltd.). The immunofluorescence was taken with a laser confocal microscope (TCS SP8, Leica), and brightfield images were taken with an Olympus microscope (BX61). The number of c‐fos^+^ neurons of the entire DR were counted manually from 3 sections per mouse.

### 2.9 Statistical analysis

Data are presented as means ± SEM. Number of experimental replicates (*n*) is indicated in each figure legend. Statistical comparisons were performed using Prism (version 8.0) with appropriate methods. Data were first tested by Kolmogorov–Smirnov test for normality and lognormality test, and data do not exhibit a normal distribution are analyzed via a non‐parametric equivalent. Data of seizure stage and after‐discharge duration in the kindling model were tested by two‐way ANOVA followed by Dunnett's comparison test or Turkey's multiple comparisons test for multiple comparisons. Data of number of stimulations in each stage were tested by one‐way ANOVA with Dunnett's comparison test or Turkey's multiple comparisons test for multiple comparisons. Number of stimulations in stage 0 was test by Kruskal–Wallis test with Dunn's comparisons test. Data of behavior test of depressive‐like behavior were tested by one‐way ANOVA with Dunnett's multiple comparison test. No statistical methods were used to pre‐determine sample size. For all analyses, a two‐tailed *p* < 0.05 was considered statistically significant.

## 3 RESULTS

### 3.1 DR 5‐HTergic neurons are activated during hippocampal seizures

**FIGURE 1:**
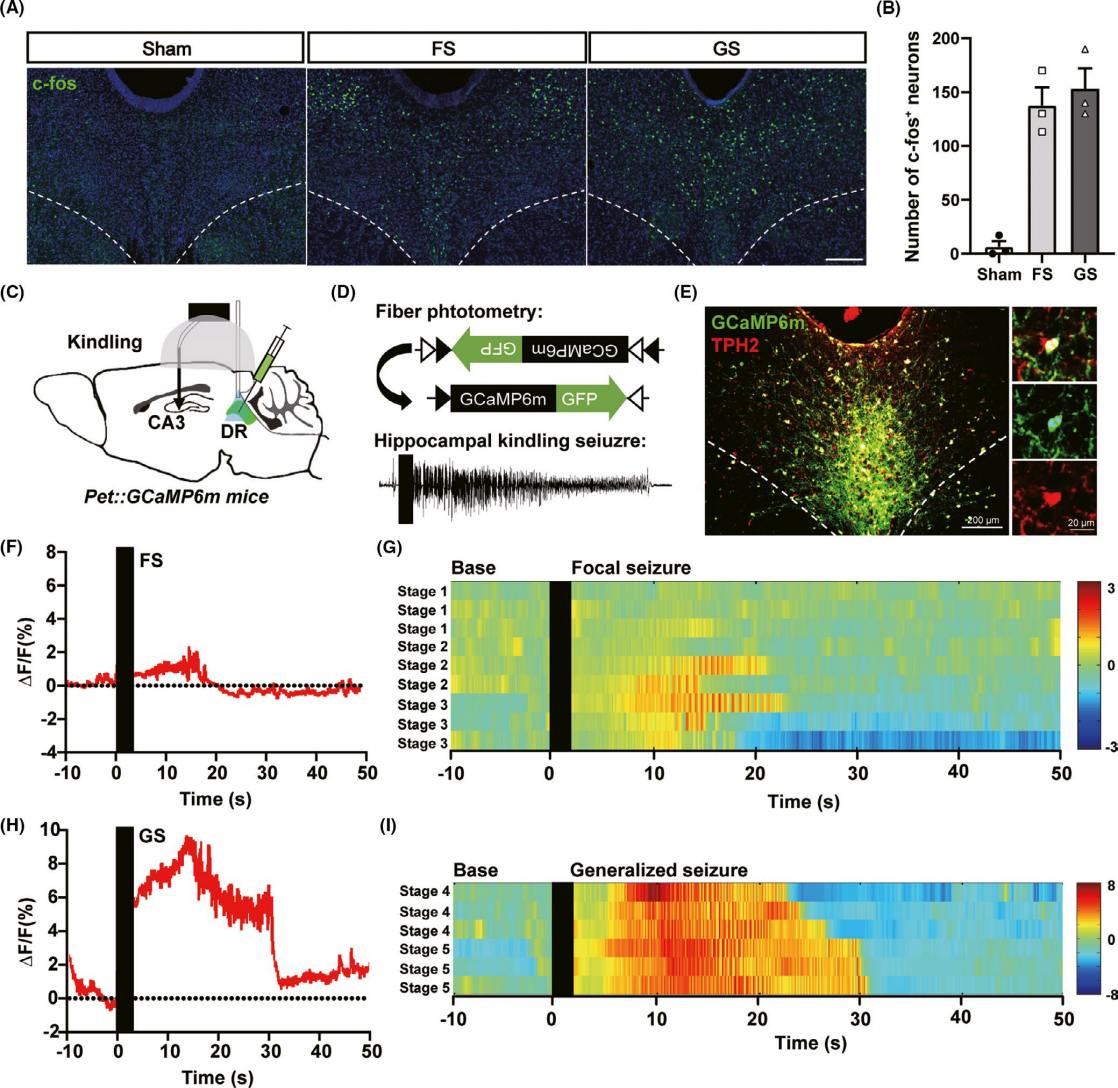
Dorsal raphe 5‐HTergic neurons are activated during hippocampal seizures. (A) Representative images of c‐fos (green) immunoreactivity in the DR in mice of focal seizure (FS) and generalized seizure (GS) 1.5 h after the last stimulation (Scale bar 200 μm). (B) Number of c‐fos^+^ neurons in the DR increased during kindling acquisition (Sham: 6.3 ± 5.4 cells/section, FS: 137.7 ± 16.9, GS: 153.3 ± 18.6, *n* = 3 mice). (C) and (D) Scheme of experiment for fiber photometry of 5‐HTergic neurons of the DR in hippocampal kindling seizures. AAV with Cre‐dependent GCaMP6m is injected into the DR of *Pet‐cre* mice. (E) Overlap of GCaMP6m (green) expression and TPH2 (red) immunoreactivity in the DR (Scale bar 200 μm). (F) and (H) Representative responses of 5‐HTergic neurons during a focal seizure (F) or a generalized seizure (H). Time 0 is aligned to the start of kindling. (G) and (I) Heatmaps of calcium signals in focal seizure (G) and generalized seizure (I). Each row represents the typical calcium signal during different stages (*n* = 3 mice). Color scale indicates the ΔF/F

To study the activity of DR neurons during hippocampal seizure, we first immunolabeled for c‐fos in the DR in mice of different seizure stages 1.5 h after the last kindling stimulation. The number of c‐fos^+^ neurons significantly increased after FS and GS (6.333 ± 5.364 cells/section in sham, 137.7 ± 16.90 cells/section in FS group, 153.3 ± 18.56 cells/section in GS group; Figure 1A,B). To directly study the activity of DR 5‐HTergic neurons during hippocampal seizure, we injected an AAV encoding the Ca^2+^ indicator GCaMP6m (AAV2/9‐Ef1α‐DIO‐GCaMP6m) into the DR of *Pet‐cre* mice (Figure 1C,D). Three weeks post‐injection, plenty of GCaMP6m was expressed in the soma of the DR and mostly colocalized for tryptophan 5‐hydroxylase 2 (TPH2), a critical enzyme in synthesis of 5‐HT (Figure 1E). After surgery and 1‐week recovery, we monitor the Ca^2+^ activity of DR 5‐HTergic neurons during kindling seizures. Consistent with the results of c‐fos immunoactivity, activity of DR 5‐HTergic neurons also significantly increased after kindling stimulation in both FS and GS (Figure 1F,H). The calcium activities increased accompanied with the increase of seizure stages (Figure 1G,I). The above results imply that DR 5‐HTergic neurons are remarkably activated during kindling seizures.

### 3.2 Optogenetic inhibition of DR 5‐HTergic neurons retards the acquisition of hippocampal seizure

**FIGURE 2:**
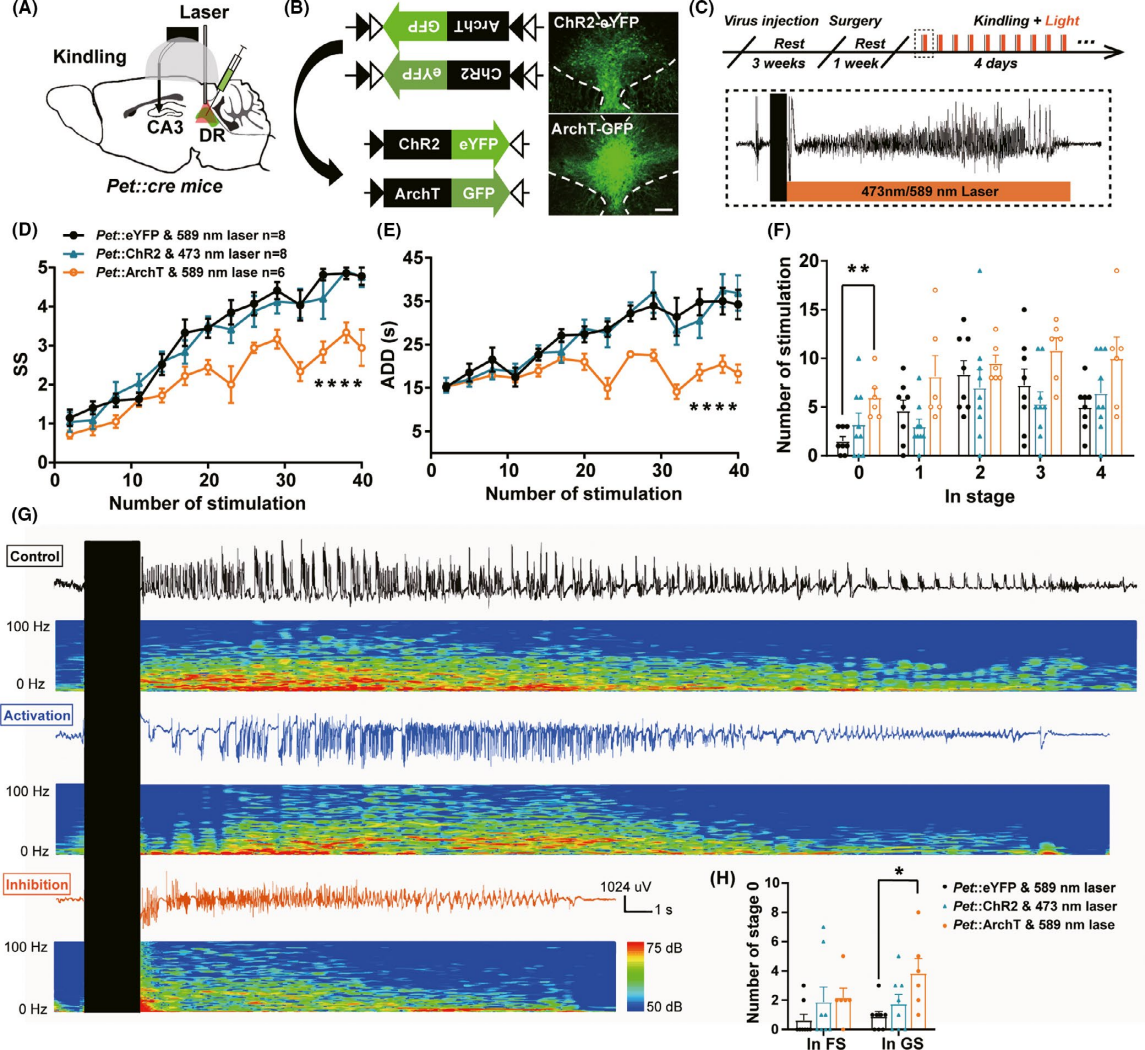
Optogenetic inhibition of DR 5‐HTergic neurons significantly retards the acquisition of hippocampal seizure. (A) Experimental schematic diagram of the hippocampal kindling model and optogenetic modulation (activation or inhibition). (B) Expression of the cre‐dependent optogenetic (ChR2 or ArchT) virus in the DR of *Pet‐cre* mice (Scale bar 200 μm). (C) Experimental scheme of optogenetic modulation during kindling acquisition. Light (488 or 593 nm) stimulation was delivered immediately after each kindling stimulation. (D–F) Effects of optogenetic modulation of DR 5‐HTergic neurons on the seizure stage (D, ****p < 0.0001, *F*(2, 20) = 12.90, Two‐way ANOVA with Dunnett's comparison test), after‐discharge duration (ADD, E, ****p < 0.0001, *F*(2,19) = 10.09, Two‐way ANOVA with Dunnett's comparison test), number of stimulations in each seizure stage (F, **p = 0.0081, *F*(2, 20) = 5.216, One‐way ANOVA with Dunnett's comparison test) in the hippocampal kindling model (*Pet*::eYFP & 589 nm laser, *n* = 8 mice; *Pet*::ChR2 & 473 nm laser, *n* = 8; *Pet*::ArchT & 589 nm laser, *n* = 6). (G) Representative EEGs, corresponding EEG spectra power of the 40th stimualtion of mice in control (black), optogenetic activation (Blue) and optogenetic inhibition (Yellow) groups. (H) Number of stimulations in stage 0 during focal seizure (before the 1st stage3) and generalized seizure (after the 1st stage3, *p = 0.0220, Kruskal–Wallis test with Dunn's comparisons test; *Pet*::eYFP & 589 nm laser, *n* = 8 mice; *Pet*::ChR2 & 473 nm laser, *n* = 8; *Pet*::ArchT & 589 nm laser, *n* = 6)

To assess the causal role of DR 5‐HTergic neurons in seizure, we then used optogenetics to selectively activate or inhibit DR 5‐HTergic neurons in hippocampal kindling model. Cre recombinase‐dependent excitatory opsin Channelrhodopsin (AAV2/9‐Ef1α‐DIO‐hChR2(H134R)‐eYFP) or inhibitory opsin archaerhodopsin (AAV2/9‐CAG‐FLEX‐ArchT‐GFP) were expressed in the DR of *Pet‐cre* mice with optical fibers implanted (Figure 2A,B). During hippocampal kindling acquisition, we applied 473 or 589 nm laser immediately after the kindling stimulation (Figure 2C). We discovered that photo‐inhibition of DR 5‐HTergic neurons significantly retarded the progress of seizure stage (Figure 2D) and shortened ADD (Figure 2E). This anti‐convulsive effect mainly existed during the late period of the seizure acquisition, where seizures were completely inhibited during GSs but not FSs demonstrated by the significantly increased the number of stimulations in stage 0 (Figure 2F,H). In contract, optogenetic activation of DR 5‐HTergic neurons had no effect on seizure acquisition (Figure 2D,E). Figure 2G demonstrates the representative ADDs and corresponding power spectrums of the 40th stimulation. Overall, Figure 2 demonstrates that selective inhibition of DR 5‐HTergic neurons has an inhibition on hippocampal seizure.

### 3.3 1‐Hz DBS of the DR retards the acquisition of hippocampal seizures through inhibition of DR 5‐HTergic neurons

**FIGURE 3:**
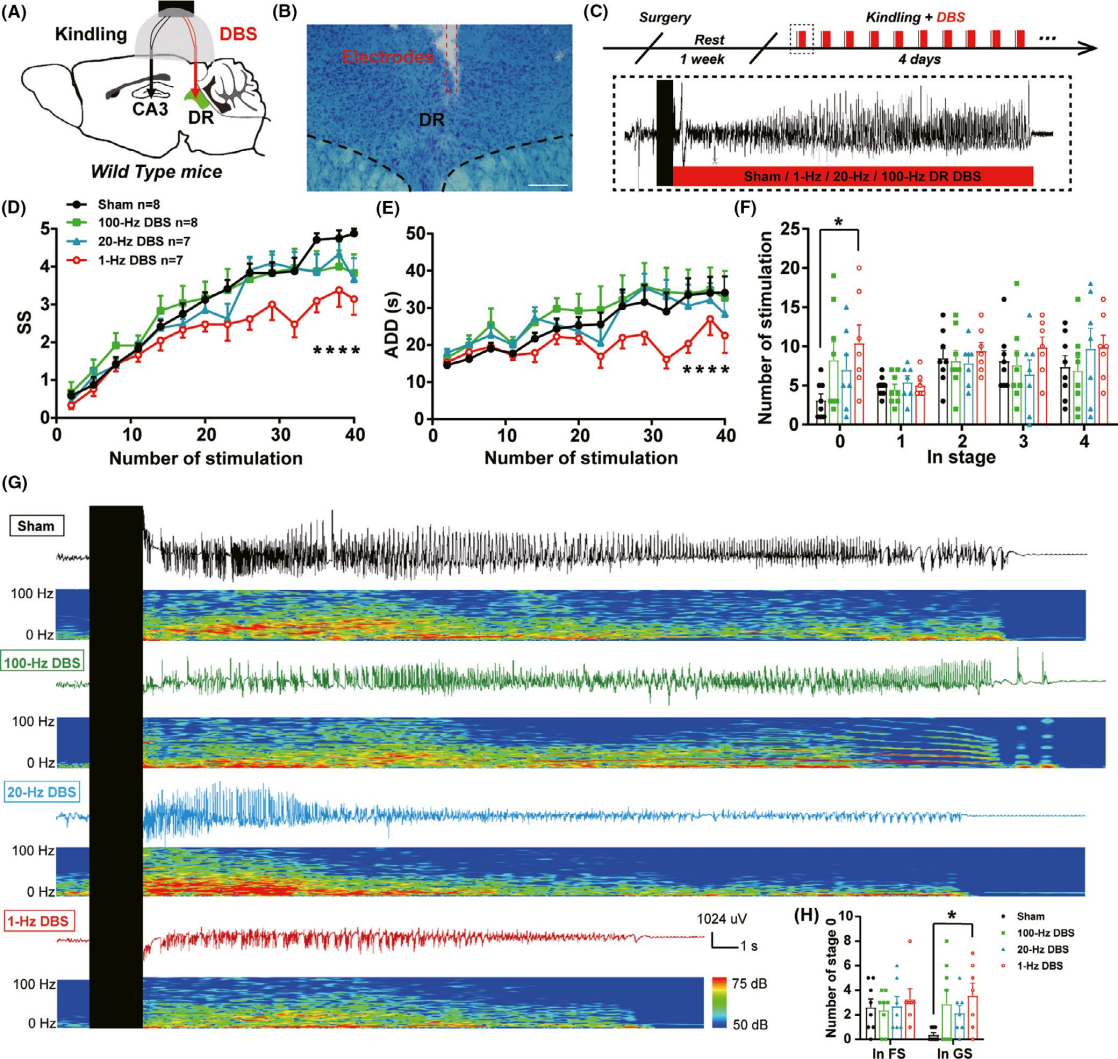
1‐Hz DBS of the DR retards the acquisition of hippocampal seizures. (A) Experimental schematic diagram of the hippocampal kindling model and the DBS of the DR. (B) Toluidine blue staining for the locations of the electrodes in the DR. (C) Experimental scheme of the DBS modulation during kindling acquisition. DBS were delivered immediately after each kindling stimulation. (D–F) Effects of the DBS of the DR on the development of seizure stage (D, ****p < 0.0001, *F*(3, 26) = 2.088, Two‐way ANOVA with Dunnett's comparison test), after‐discharge duration (ADD, E, ****p < 0.0001, *F*(3, 26) = 1.496, Two‐way ANOVA with Dunnett's comparison test), number of stimulations in each seizure stage (F, *p = 0.0345, *F*(3, 26) = 2.555, One‐way ANOVA with Dunnett's comparison test) in the hippocampal kindling model (Sham, *n* = 8 mice; 100‐Hz DBS, *n* = 8 mice; 20‐Hz DBS, *n* = 7 mice; 1‐Hz DBS, *n* = 7 mice). (G) Representative EEGs, corresponding EEG spectra power of mice in sham (black), 100‐Hz DBS (green), 20‐Hz DBS (blue) and 1‐Hz DBS (red) groups. (H) Number of stimulations in stage 0 during focal seizure (before the 1st stage3) and generalized seizure (after the 1st stage3, *p = 0.0330, Kruskal–Wallis test with Dunn's comparisons test, Sham, *n* = 8 mice; 100‐Hz DBS, *n* = 8 mice; 20‐Hz DBS, *n* = 7 mice; 1‐Hz DBS, *n* = 7 mice)

Next, we aimed to investigate whether DBS, an approach much suitable for clinical translation medicine, in the DR would have a therapeutic potential for hippocampal seizure. We implanted electrodes into the DR for the delivery of DBS and electrodes into the ventral hippocampus for EEG recording and kindling stimulation (Figure 3A–C). We found that 1‐Hz DR DBS significantly inhibited the progress of seizure stage (Figure 3D) as well as shortened ADD (Figure 3E). Similar as that of optogenetic inhibiting 5‐HTergic neuron, the anti‐seizure effect of DBS existed mainly during the late period of the kindling acquisition, where DBS significantly increased the number of stimulations in stage 0 during GSs (Figure 3F,H). Whereas, DBS at the frequency of 20 or 100 Hz had no effect on the kindling acquisition (Figure 3D–F). Representative ADDs and corresponding power spectrums were shown in Figure 3G. These data demonstrated that 1‐Hz‐specific DBS retarded the acquisition of hippocampal seizure.

**FIGURE 4:**
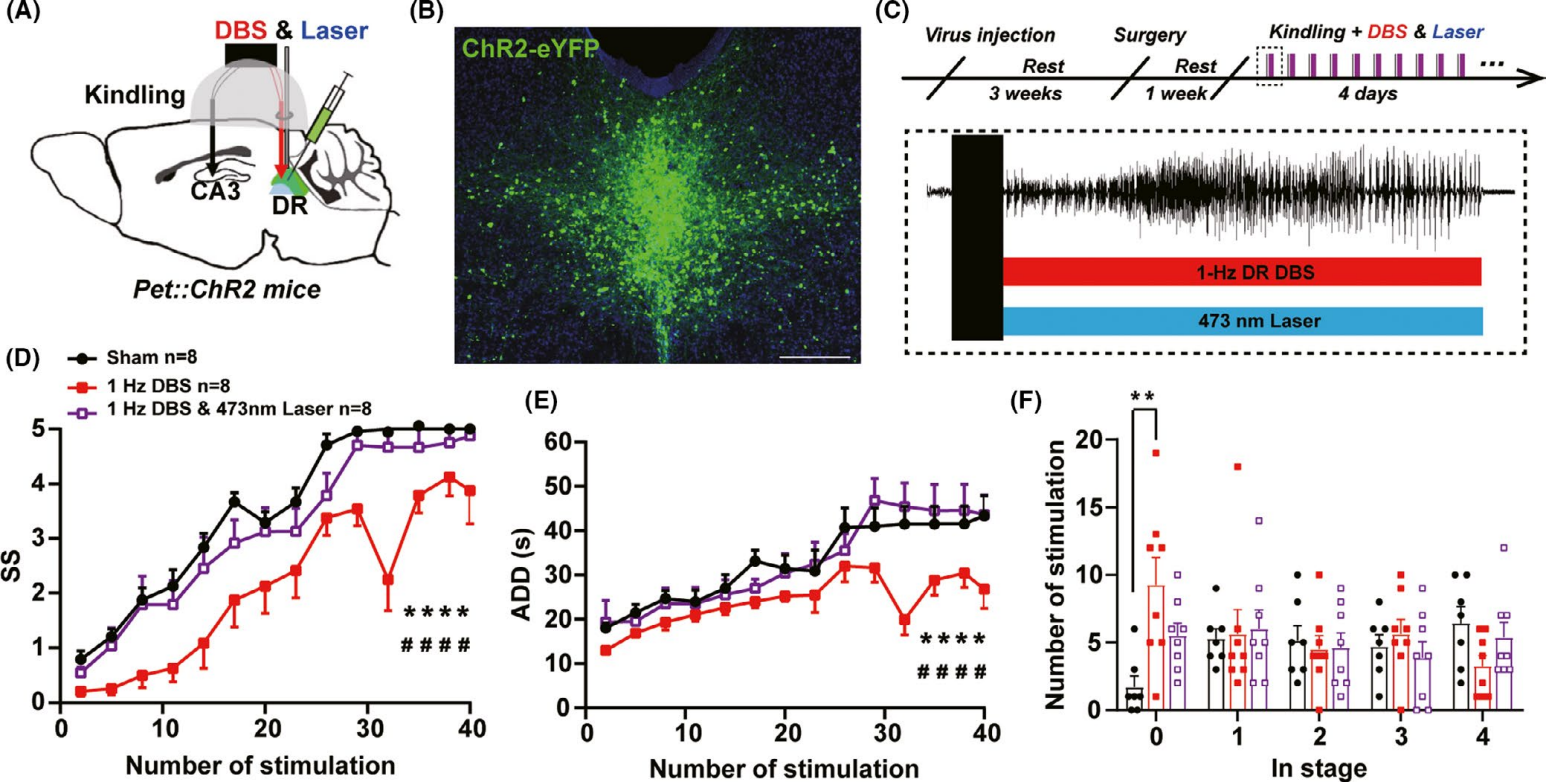
Activation of DR 5‐HTergic neurons reverses anti‐epileptic effects of 1‐Hz DBS on hippocampal seizures. (A) Experimental schematic diagram of the hippocampal kindling model, optogenetic activation and DBS. (B) Expression of the cre‐dependent ChR2 virus in the DR of *Pet‐cre* mice (Scale bar 200 μm). (C) Experimental scheme of DBS and optogenetic modulation during kindling acquisition. DBS and 473‐nm laser were delivered immediately after each kindling stimulation. (D–F) Effects of 1‐Hz DR DBS, combined with optogenetic activation of DR 5‐HTergic neurons, on the seizure stage (D, for sham vs.1‐Hz DBS: ****p < 0.0001, for sham vs. 1‐Hz DBS & 473 nm laser: ^####^ *p* < 0.0001, *F*(2, 21) = 9.819, Two‐way ANOVA with Turkey's comparison test), after‐discharge duration (ADD, E, for sham vs. 1‐Hz DBS: ****p < 0.0001, for sham vs. 1‐Hz DBS and 473‐nm laser: ^####^ *p* < 0.0001, *F* (2, 21) = 3.632, Two‐way ANOVA with Turkey's comparison test), number of stimulations in each seizure stage (F, **p = 0.0056, Kruskal–Wallis test with Dunn's comparisons test) in the hippocampal kindling model (Sham, *n* = 8 mice; 1‐Hz DBS, *n* = 8 mice; 1‐Hz DBS & 473 nm Lase, *n* = 8 mice)

As low frequency electrical stimulation may induce depotentiation or long‐term depression, to test whether 1‐Hz DBS takes effect through inhibition of DR 5‐HTergic neurons, DBS was applied simultaneously combined with activation of DR 5‐HTergic neurons. Cre recombinase‐dependent Channelrhodopsin was expressed in the DR of *Pet‐cre* mice with optical fibers implanted for laser delivery and electrodes implanted for DBS (Figure 4A,B). After 1‐week recovery, 1‐Hz DBS and 473‐nm laser were applied simultaneously in the DR during kindling seizure (Figure 4C). It is discovered that activation of DR 5‐HTergic neurons fully reversed the anti‐seizure effect of 1‐Hz DBS on seizure stage, ADD and number of stimulations in stage 0 (Figure 4D–F). Above results indicated that 1‐Hz DBS of the DR inhibits hippocampal seizure possibly through inhibition of DR 5‐HTergic neurons.

### 3.4 1‐Hz DBS of the DR has no effect on depressive‐like behavior

Since the comorbid depression is common in epilepsy and closely interrelated with the activity of DR neurons,^36^, ^38^ we tested whether 1‐Hz DBS of the DR would attenuate depressive‐like behavior. We applied sucrose preference test (SPT), splash test (ST), and tail suspension test (TST) in mice after kindling acquisition and 1‐Hz DR DBS (Figure S1A), as SPT and ST tests usually reflect anhedonia‐like behavior while TST test indicates despair‐like behavior in depression.^39^ It is demonstrated that neither kindling acquisition nor 1‐Hz DR DBS had any effect on depressive‐like behavior in SPT, ST, and TST (Figure S1B–D).

## 4 DISCUSSION

Kindling‐induced seizures are associated with activation of pro‐inflammatory pathways^40^ and growth of mossy fibers,^41^ which are known to contribute to epileptogenesis. However, the causal role of the DR 5‐HTergic neurons in kindling model of TLE is still unknown. The present study demonstrates that the 5‐HTergic neurons in the DR are remarkably activated during seizure and selective inhibition of the DR 5‐HTergic neurons significantly retards the development of the hippocampal kindling seizure in mice. For clinical translation, we found that 1‐Hz DBS, but not 20‐Hz or 100‐Hz DBS, of the DR also has the anti‐seizure effect (possibly through inhibition of hyperactivity of DR 5‐HTergic neurons) without affecting depressive‐like behavior. Together, these results support important role of the DR 5‐HTergic neuron in epilepsy, and inhibition of hyperactivity of DR 5‐HTergic neurons may present promising anti‐seizure effect.

### 4.1 Neuron activities of the DR in epilepsy

Activities of the DR were reported to be variable largely with increased, decreased, and not changed during seizure in different epilepsy models.^23^, ^24^, ^25^ It should be noted that all of these researches were applied in anesthetized or decapitated animals. In awake *WAG/Rij* rat model of absence epilepsy, spike and wave discharges propagate to the DR with a short delay with increased firing rate of the DR neurons.^25^ However, in a same absence epilepsy model, level of 5‐HT metabolite 5‐hydroxyindoleacetic acid (5‐HIAA) in the thalamus was decreased and a significant negative correlation between the severity of epilepsy and the thalamic levels of 5‐HT has been observed in epileptic *WAG/Rij* rats.^42^ What happened indeed to the activities of the DR and DR 5‐HTergic neurons during hippocampal seizure is still unclear. Here, we found that in hippocampal kindling seizure of awake mice, overall c‐fos expression and activities of 5‐HTergic neurons are both significantly increased during hippocampal seizure. The calcium activities increased along with the seizure stages and make more remarkable increase during the GS than during FS. It is indicated that DR 5‐HTergic neurons take different degrees of participation in different periods of epilepsy, and may have more important functions in GS.

### 4.2 Optogenetic modulation of DR 5‐HTergic neurons in epilepsy

In early studies, electrolytic lesion of the midbrain raphe and systematic lesion of the 5‐HTergic system significantly increase the electrographic seizure activity while systematic enhancing the 5‐HTergic system inhibit the seizure.^8^ In addition, 5‐HT1A/7 receptor or 5‐HT2C receptor agonist significantly reduced epileptic discharges induced by maximal dentate activation in a partial complex model of TLE.^43^, ^44^ It should be noticed that these intervention studies modulate the whole brain 5‐HTergic system without taking the heterogeneous function of the 5‐HTergic sub‐system into consideration.^37^, ^45^ Notably, conflicting reports about the role of SSRIs in epilepsy indicates both anti‐seizure and pro‐seizure results.^14^, ^15^, ^46^, ^47^ It indicates that precise investigation of the role of the 5‐HTergic subsystems in epilepsy is necessary. Interestingly, in hippocampal kindling model, we found that selective optogenetic inhibition, but not activation, of DR 5‐HTergic neurons significantly retard the acquisition of the seizure. This result seems to be not consistent with the traditional role of the 5‐HTergeic system in epilepsy. The possible reasons are listed as below aspects:

1. Time‐aligned modulation. The anti‐seizure effects are achieved by the manner of mimicking close‐loop optogenetic inhibition of the hyperactivity of DR 5‐HTergic neurons, suggesting the participation of DR 5‐HTergic neurons as endogenous mechanism in seizure. Not unexpectedly, optogenetic activation of DR 5‐HTergic neurons (already hyperactivated during seizure) has no effect on the seizure acquisition due to the ceiling effect.
2. The 5‐HTergic subsystems may play different roles in different periods of epilepsy. In our study, the anti‐seizure effect of inhibiting DR 5‐HTergic neurons shows at the late period of the seizure acquisition. One explanation is that functions of DR 5‐HTergic neurons may be more important in the late period of epilepsy, since calcium activities also demonstrated that degrees of participation of DR 5‐HTergic neurons are higher during GS than during FS. Another explanation is the intrinsic characteristics of the central 5‐HTergic system that SSRIs often take time to make an effect and acute intervention hardly play a role.^47^
3. The 5‐HTergic subsystems may play different roles in different models of epilepsy. In a DBA/1 mouse model of epilepsy, optogenetic activation of DR 5‐HTergic neurons significantly suppresses respiratory arrest but has limited effect on acoustic stimulation‐induced seizure intensity or pentylenetetrazole‐induced GS.^28^ However, the modulation of 5‐HTergic system in epilepsy may be seizure type–dependent. In hippocampal kindling model of TLE, another type of FS with secondary GS, activities of DR 5‐HTergic neurons increased aberrantly during hippocampal seizures. Overall, DR 5‐HTergic system plays a critical role in epilepsy, but more precise modulation of DR 5‐HTergic neurons and circuits need to be studied in different models of epilepsy.

### 4.3 DBS of the DR in epilepsy

DBS is developed into an optional treatment for medication‐refractory epilepsy and depression, although the optimal target and stimulation are still unclear. In this research, we discovered that DBS of the DR with the frequency of 1 Hz, but not 20 or 100 Hz, could mimic the anti‐seizure effect of the inhibition of DR 5‐HTergic neurons that significantly inhibit the seizure during the late period of the kindling acquisition. As low‐frequency electrical stimulation of neurons may induce depotentiation or long‐term depression,^48^ it is likely that 1‐Hz DR DBS is implicated in the depotentiation or inhibition of 5‐HTergic neurons and thus produce anti‐seizure effects. To confirm this hypothesis, activation of DR 5‐HTergic neurons fully reverse the anti‐seizure effect of 1‐Hz DR DBS, which implied the participation of DR 5‐HTergic neurons in the mechanism of 1‐Hz DR DBS. In addition, modulation of the DR and its 5‐HTergic systems is widely reported to have an effect on depressive‐like behaviors. Acute or chronic activation of DR 5‐HTergic neurons induce an antidepressive‐like effect,^49^, ^50^ but inhibitions result in different consequences including increased floating behavior in forced swim test^51^ or no effect.^52^ In the present study, we found that 1‐Hz DBS of the DR during kindling seizures has no effect on depressive‐like behaviors in SPT, SP, and TST in epileptic mice. This can be due to the different timing of DBS modulation compared with previous study, in which DBS is applied during the behavior test, indicating that seizure‐aligned stimulation of the DR does not affect the depressive‐like behaviors of epileptic mice during inter‐ictal period.

In conclusion, we found that DR 5‐HTergic neurons are remarkably activated during seizure, and optogenetic inhibition of DR 5‐HTergic neurons significantly retards the acquisition of hippocampal seizure. Further, 1‐Hz DR DBS similarly inhibit​ed hippocampal seizure which was blocked by the activation of DR 5‐HTergic neurons. Together, inhibition of hyperactivity of DR 5‐HTergic neuron may present promising anti‐seizure effect and the DR is a potential target for the treatment of epilepsy.

## CONFLICT OF INTEREST

None.

## Supporting information

Fig S1

## Data Availability

The data that support the findings of this study are available from the corresponding author upon reasonable request.
