# Supplementary material for: Inhibition of hyperactivity of the dorsal raphe 5‐HTergic neurons ameliorates hippocampal seizure
Source: CNS Neurosci Ther. 2021 May 6;27(8):963–72. doi: 10.1111/cns.13648 (PMC8265946; doi:10.1111/cns.13648)
Supplement: Supplementary file 1 — Fig S1 [file CNS-27-963-s001.docx]

**
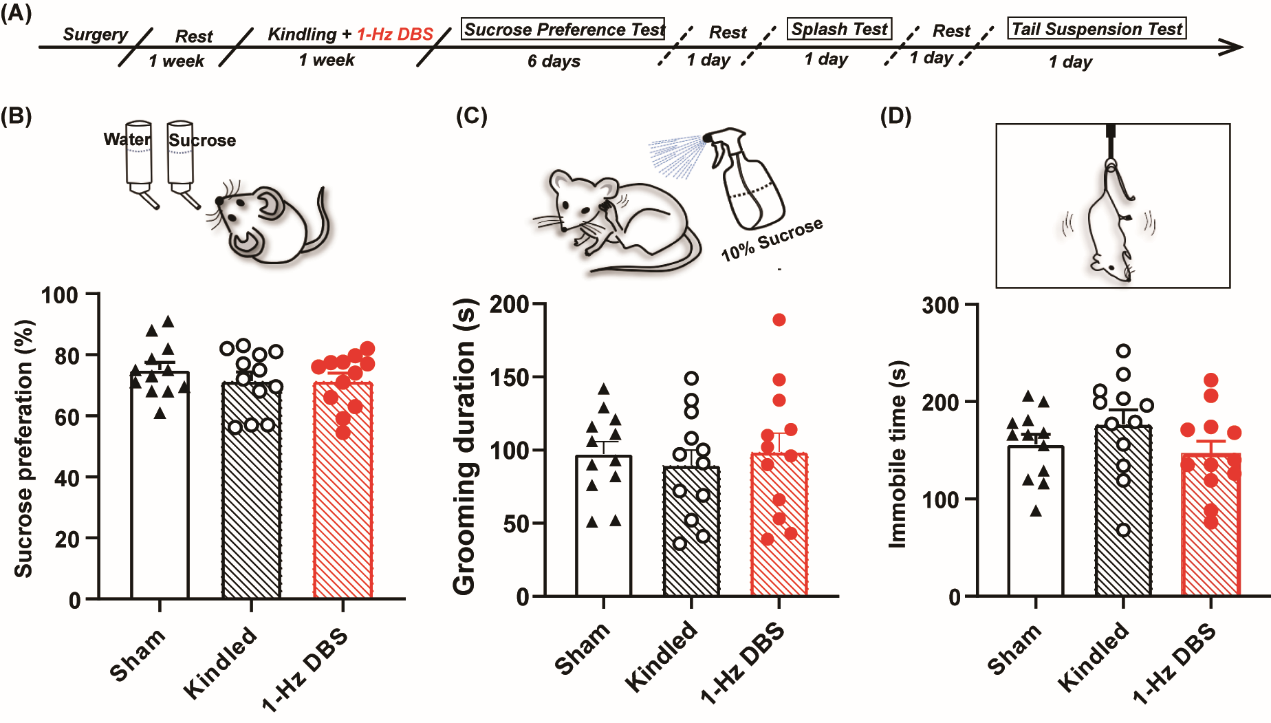
**

**FIGURE. S1**  1-Hz DBS of the DR has no effect on depressive-like bahavior. (A) Experimental scheme diagram of the 1-Hz DBS of the DR in hippocampal kindling mice and the behavior tests (sucrose preference test, splash test, tail suspension test) before and after kindling. (B) 1-Hz DBS of the DR has no effect on sucrose preference of kindled mice in sucrose preference test (SPT, n=12 mice). (C) 1-Hz DBS of the DR has no effect on the grooming duration of hippocampal kindled mice in splash test (ST, n=12 mice). (D) 1-Hz DBS of the DR has no effect on the immobile time in forced swim test (FST, n=12 mice).
